# Combined caudal retrocostal and lateral ultrasound-guided approach for transversus abdominis plane injection: A descriptive pilot study in pig cadavers

**DOI:** 10.1371/journal.pone.0248131

**Published:** 2021-03-10

**Authors:** Ivana Calice, Silvio Kau, Christian Knecht, Pablo E. Otero, M. Paula Larenza Menzies

**Affiliations:** 1 Clinical Unit of Anesthesiology and Perioperative Intensive-Care Medicine, Department for Small Animals and Horses, Vetmeduni Vienna, Vienna, Austria; 2 Institute of Topographic Anatomy, Department of Pathobiology, Vetmeduni Vienna, Vienna, Austria; 3 Department for Farm Animals and Veterinary Public Health, Vetmeduni Vienna, University Clinic for Swine, Vienna, Austria; 4 Department of Anesthesiology, Faculty of Veterinary Medicine, University of Buenos Aires, Buenos Aires, Argentina; University of Bari, ITALY

## Abstract

Transversus abdominis plane (TAP) block is a regional anesthetic technique used to desensitize the abdominal wall in several species. This study aimed to describe the anatomical characteristics of the abdominal wall and to identify a feasible approach for an US-guided TAP injection that would result in adequate staining of the relevant nerves in the abdominal wall in pig cadavers. Fresh cadavers from five Landrace pigs (age, 12 weeks; body weight, 35.5 ± 1.6 kg) were used. One pig (n = 1) was anatomically dissected, and four pigs (n = 4; i.e., 8 hemiabdomens) were used for TAP injections and evaluation of dye spread. The volume of 0.3 mL/kg/injection point of methylene blue was injected bilaterally. In the caudal retrocostal approach, the injection was performed ventral to the most caudal part of the costal arch. In the lateral approach, the injection was performed between the last rib and iliac crest. A needle was inserted in plane for the caudal retrocostal and the lateral approach caudocranially and craniocaudally, respectively. Successful staining was defined as presence of dye on the nerve for a length of >1 cm in its entire circumference. The TAP was found between different muscle layers in the described anatomical regions. In the caudal retrocostal approach the TAP was found between the external abdominal oblique and transversus abdominis muscle bellies. In the lateral approach the TAP was found between the internal abdominal oblique and transversus abdominis muscles. The approach combining lateral and caudal retrocostal injections at the studied volume stained a median of 5 (3–6) target nerves from the fourth-last thoracic nerve to L2 (six nerves). Combined caudal retrocostal and lateral TAP injections of 0.3 mL/kg/injection point, resulted in staining of target nerve branches which supply the periumbilical and caudal abdominal wall in pig cadavers.

## Introduction

Regional anesthesia is a widely used technique in human and veterinary medicine [1]. It provides numerous benefits in the minimization of adverse effects over other techniques such as the use of systemic analgesics [1, 2]. Additional advantages of regional anesthesia may be attained in pigs undergoing surgery, when administration of systemic analgesics (e.g., opioids) is usually not licensed [3, 4]. Moreover, when pigs are used as a research models, in skin, liver and kidney transplantation surgery [5–7], non-adequate analgesia as well as the use of non-steroidal analgesics and opioid drugs may bias research results [4, 8].

The ultrasound-guided transversus abdominis plane (TAP) block is a regional anesthetic technique used in humans to desensitize the anterolateral abdominal wall, which corresponds to the ventrolateral abdominal wall in veterinary patients [9, 10]. It involves the injection of local anesthetic into the fascial plane between the transversus abdominis (TAM) and either the internal oblique (IOM) or the rectus abdominis (RAM) muscles, where the ventral branches of the thoracolumbar spinal nerves are located [11–13]. Based on anatomical similarities observed in the innervation of the abdominal wall between species [9–13], this technique has been extrapolated to animals undergoing abdominal surgery (e.g., dog, cat, horse, calf, lynx, chinchilla) [14–19]. Further development of this block has occurred in veterinary medicine as anatomy of the abdominal wall differs between the species. A single-entry approach may be sufficient for certain indications and species [19], while in other circumstances a double-entry approach may be required e.g., lateral and retrocostal [14, 20, 21]. Despite the popularity of the TAP block and the commonly performed abdominal surgical procedures in pigs, either for clinical or research purposes, this technique has not yet been investigated in this species.

This pilot study aimed i) to describe the anatomical characteristics of the abdominal wall, ii) to identify an adequate injection site for an US-guided in-plane TAP injection and iii) to determine the distribution pattern of a 0.3 mL/kg/injection point dye solution after caudal retrocostal and lateral TAP injections in pig cadavers. The hypothesis of the present study was that a two-point (caudal retrocostal and lateral) TAP injection approach would be required to adequately stain all the relevant nerves of the abdominal wall.

## Materials and methods

### Animals and study design

Fresh cadavers from five (n *=* 5) female Landrace pigs (12 weeks of age; mean bodyweight ± SD = 35.5 ± 1.6 kg) that had been euthanized by intravenous injection of T61 (Merck Sharp & Dohme, NJ, US) under general anesthesia with sevoflurane (Sevorane; Abbot Ltd., Austria) entered the study.

The pigs were euthanized 30 minutes prior to this investigation as part of an unrelated non-survival study, which did not involve thoracoabdominal procedures and was approved by the ethics committee of the Austrian Federal Ministry of Education, Science and Research; permit number: BMBWFW-68.205/0135-WF/V/3b/2014. The present cadaveric study complied with the guidelines specified by the local ethics committee of the University of Veterinary Medicine Vienna for the maintenance of good scientific practice and did not require a further permission for animal experimentation.

The study was performed in two phases. In phase I, one cadaver (n *=* 1) was dissected to describe the anatomy of abdominal wall muscles and the segmental nerves. Additionally, suitable sites for caudal retrocostal and lateral TAP injections were determined. In phase II, four cadavers (n *=* 4; i.e., 8 hemiabdomens) were used to evaluate the injectate distribution pattern after caudal retrocostal and lateral TAP injections. Each injection was performed on both sides of the abdomen (in random order) of each cadaver.

### Phase I: Anatomical assessment

**Fig 1:**
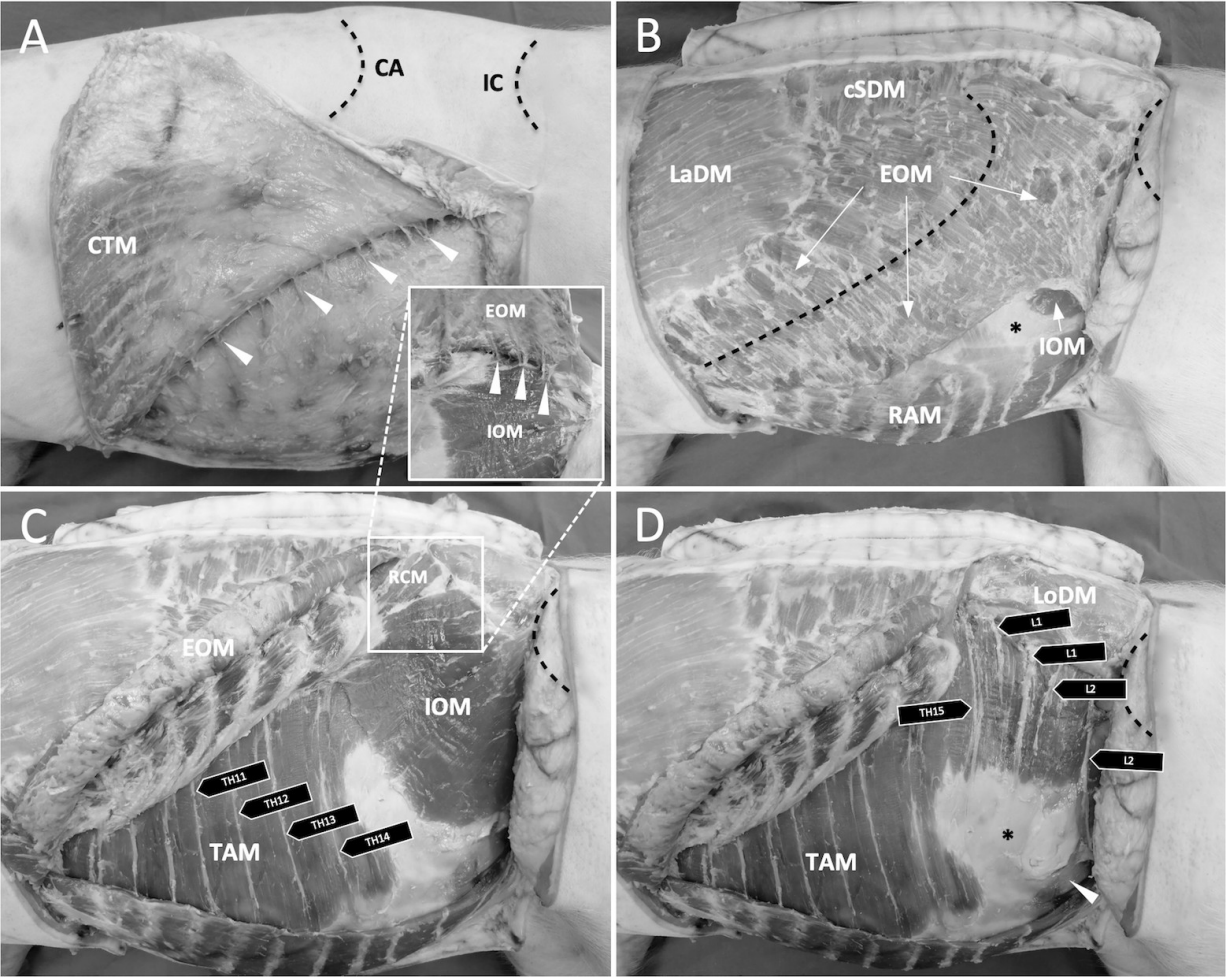
Relevant anatomy of the ventrolateral abdominal wall muscles and thoracolumbar segmental nerve branches. The left hemiabdomen of a pig with 15 costae and six lumbar vertebrae in right lateral recumbency. (**A**) Dissection of skin and cutaneous trunci muscle (CTM). The costal arch (CA) and iliac crest (IC) are indicated by dotted lines. Arrowheads indicate neurovascular bundles of lateral branches from the ventral rami of segmental nerves that perforate the abdominal wall and enter the CTM and overlying skin. Similarly, bundles of nerves (i.e., short branches of Th_15_, L_1_ and L_2_ in this case) and blood vessels were seen in the paralumbar region perforating the internal abdominal oblique muscle (IOM) and entering the external abdominal oblique muscle (EOM) and overlying skin (insert). (**B**) After removal of skin, skin muscle, fat and fascial elements the latissimus dorsi muscle (LaDM), caudal serratus dorsalis muscle (cSDM), EOM and rectus abdominis muscle (RAM) were visualized. Beneath the aponeurosis of the EOM (removed), ventral aspects of the IOM and its aponeurosis (asterisk) emerged. (**C**) Visualization of caudal retrocostal TAM and thoracic segmental nerve branches (Th_11–14_) running on it and IOM after removal of EOM and fascial elements. RCM, retractor costae muscle. (**D**) Visualization of paralumbar TAM and segmental nerve branches of the last thoracic nerve (Th_15_) and lumbar nerves (L_1–2_) running on it after removal of IOM and RCM. LoDM, longissimus dorsi muscle. Characteristic TAM aponeurosis (asterisk) and caudo-ventral parietal peritoneum (arrowhead).

In the cadaver assigned to anatomical dissection, a ventral midline skin incision of the abdomen was performed. The skin and the cutaneous trunci muscle (CTM), which covers the lateral wall of abdomen and thorax, were separated from the regions of interest. Then, the external abdominal oblique muscle (EOM) was separated from its ventral and caudal insertion aponeurosis and everted craniodorsally to visualize the IOM, retrocostal parts of the TAM and branches of thoracic spinal nerves running over the TAM. The IOM was separated from its cranial and ventral insertion aponeurosis and reverted caudo-dorsally to visualize paralumbar parts of the TAM and related thoracolumbar nerves. The relevant anatomical structures can be visualized in Fig 1.

The relevant nerves for the phase II of the study were the medial branches of the ventral rami of the last three to four thoracic and first two to three lumbar spinal nerves. Since the number of thoracic and lumbar nerves depends on the number of thoracic and lumbar vertebrae, for the purpose of this study the last thoracic nerves were named from caudal to cranial as the last (costoabdominal), second-last, third-last thoracic nerves and so on. The lumbar nerves were named respectively after the number of the lumbar segment from which they originate. The last two thoracic and the lumbar (L_1-6(7)_) nerves were tracked back to their respective intervertebral foramen by an anatomist (SK). The segmental origin and characteristics of lumbar nerves were investigated in detail in the pig used in phase I with six lumbar vertebrae, and in one pig with seven lumbar vertebrae. The best potential needle accesses site for caudal retrocostal and lateral approach were determined.

### Phase II: TAP injections and dye spread evaluation

**Fig 2:**
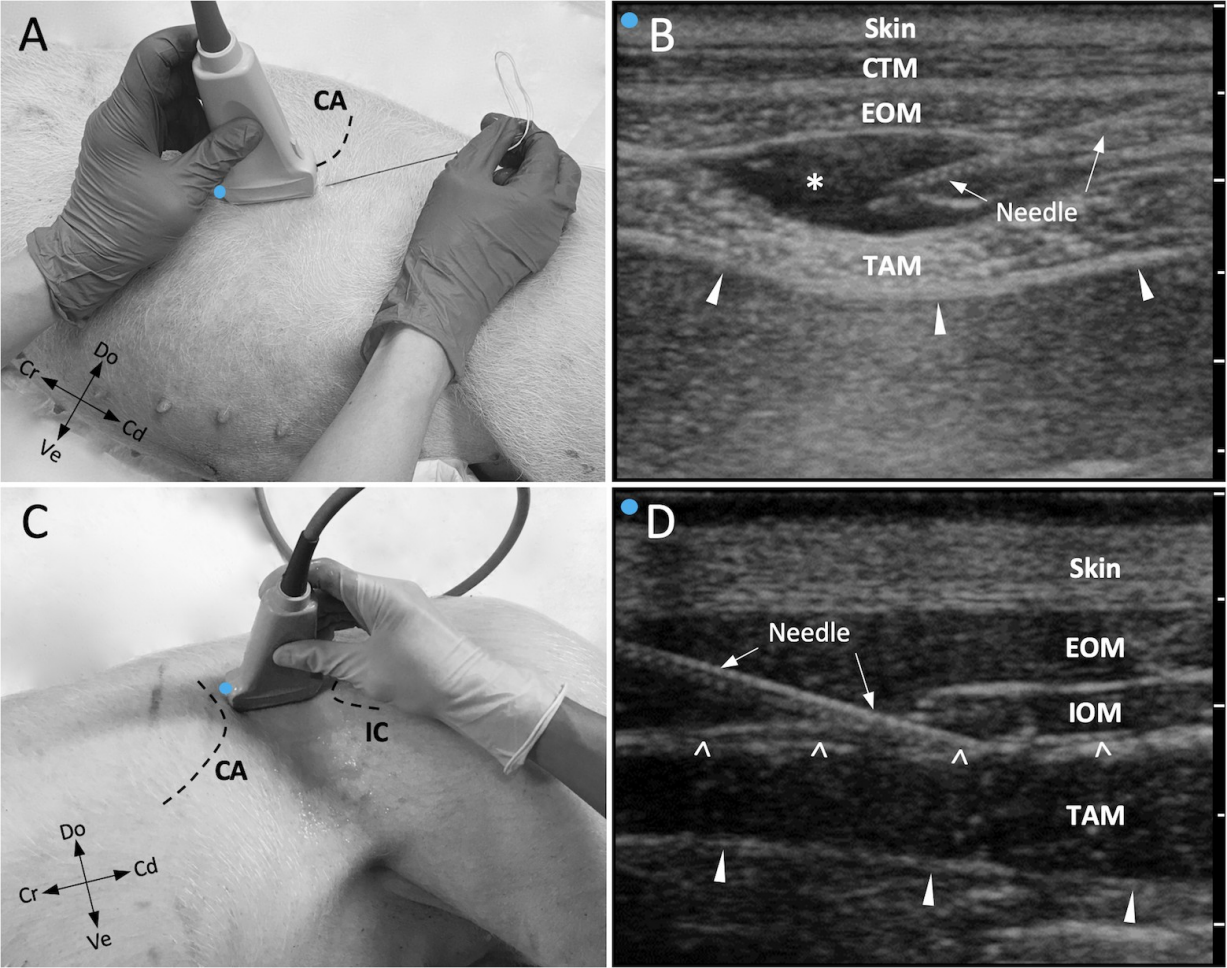
Ultrasound transducer positioning and ultrasound imaging during double-entry Transversus Abdominis Plane (TAP) injections. **(A)** Caudal retrocostal approach to the TAP. The ultrasound transducer is placed parallel and immediately ventral to the costal arch. Do, dorsal; Cd, caudal; Ve, ventral; Cr, cranial; CA, costal arch. **(B)** Corresponding ultrasound image of muscular layers forming caudo-lateral retrocostal aspects of the abdominal wall in pigs. The left side of the image corresponds to the side of the transducer marked with an indicator (blue). CTM, cutaneous trunci muscle. The parietal peritoneum is indicated by arrowheads. The needle is inserted in the fascial plane between the external abdominal oblique muscle (EOM) and transversus abdominis muscle (TAM). Injectate causing hydrodissection of fascial plane (asterisk). Markers on the far right indicate depth markers (1 cm). **(C)** Lateral approach to the TAP. The ultrasound transducer is positioned in the paralumbar fossa, parallel to the long axis of the body. Do, dorsal; Cd, caudal; Ve, ventral; Cr, cranial; CA, costal arch; IC, iliac crest. **(D)** Corresponding ultrasound image of muscular layers forming caudo-dorsal lateral aspects of the abdominal wall in the pig. The left side of the image corresponds to the side of the transducer marked with an indicator (blue). IOM, internal abdominal obliquus muscle. The parietal peritoneum is indicated by arrowheads. The ultrasound needle is inserted in the fascial plane (open arrowheads) between IOM and TAM. Markers on the right of image indicate depth markers (1 cm).

Ultrasonography of the targeted injection points was performed by a proficient US operator. A 6–13 MHz linear transducer (linear US transducer MicroMaxx, Fujifilm SonoSite, WA, USA) attached to an US machine (MicroMaxx, Fujifilm SonoSite, WA, USA) and echogenic needles (21 gauge x 100 mm; SonoPlex STIM cannula; Pajunk, Germany) were used. The cadavers were positioned in lateral recumbency with the side to be injected uppermost. In all the cases, the needle was always introduced using an in-plane technique (along the axis of the US-probe). A volume of 0.3 mL/kg/injection point of a 1% methylene blue solution (Methylene Blue 1% w/v aq. soln., Alfa Aesar, ThermoFischer GmbH, Germany) was applied in all injections. For described double-entry approach to the TAP, the lateral injection was performed before the caudal retrocostal injection in all hemiabdomens (Fig 2).

#### Caudal retrocostal TAP injection

After palpating the costal arch and the last rib, the skin of the area was cleaned. Alcohol gel (Softa-Man, ViscoRub, B. Braun, Austria) was then applied to facilitate acoustic coupling. The US transducer was placed parallel to the costal arch in line with the last rib, with the mark facing cranially (Fig 2A). The final position of the transducer was the one that allowed the recognition of the following structures: parietal peritoneum, TAM, EOM, CTM, skin (profound to superficial structures) (Fig 2B). Then, the needle was inserted caudo-cranially with a 20°–30° angle to the skin surface and advanced towards the target plane. When the tip of the needle was identified in the TAP after visualization of its entire shaft, a staining test solution (1 mL) was injected to confirm correct positioning by hydrodissection of the facial plane (Fig 2B). In case of incorrect spread of the test solution (e.g., intramuscular spread or wrong interfascial plane) the needle was repositioned and another test dose applied. This procedure was repeated until the test volume was observed to be injected in the target fascial plane, after which the predefined volume was injected.

#### Lateral TAP injection

The skin of the paralumbar fossa was cleaned and alcohol gel was applied to facilitate acoustic coupling. The US transducer was positioned parallel to the long axis of the body caudally to the last rib and cranially to the iliac crest, with the mark probe facing cranially (Fig 2C). The US transducer was then glided slowly over the abdominal wall toward its ventral region to identify the relevant sono-anatomical reference structures such as the parietal peritoneum, TAM, IOM, EOM and skin (profound to superficial structures) (Fig 2D). The optimal US window for the TAP injection was selected upon clear visualization of the target interfascial plane. The needle was inserted just caudal to the last rib, cranio-caudally with a 20°–30° angle to the skin surface and advanced in a cranio-caudal direction towards the TAP. Injection of test volumes and predefined volume within the TAP were performed as described for the caudal retrocostal approach.

### Dye spread evaluation

**Fig 3:**
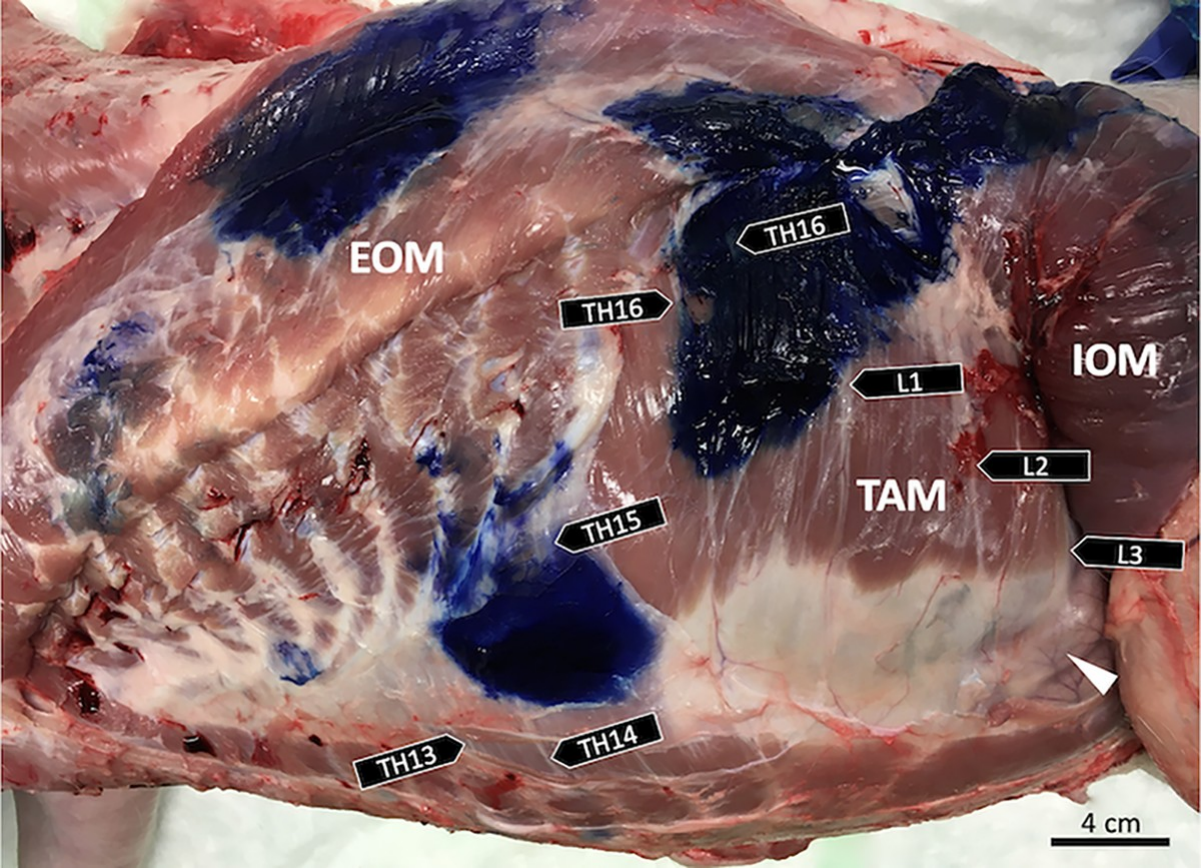
Dissection of the left hemiabdomen abdominal wall and thoracolumbar segmental nerve branches following ultrasound-guided double-entry TAP injection. The pig had 16 thoracic and seven lumbar vertebrae. Injection of 0.3 mL/kg of 1% methylene blue solution in the lateral and caudal retrocostal TAP space resulted in positive staining from the third-last thoracic (Th_14_) to the second lumbar (L_2_) nerve. The upper left stained area was evident after eversion of the external abdominal oblique muscle (EOM). IOM, internal abdominal oblique muscle; TAM, transversus abdominis muscle; caudo-ventral parietal peritoneum (arrowhead); left, cranial; right, caudal.

Anatomical dissection of the hemiabdomens was performed ten minutes after the retrocostal injection, as described in Phase I. The staining of medial branches of the ventral rami of thoracolumbar spinal nerves with methylene blue was evaluated with direct visualization (Fig 3). A positive nerve staining was defined as more than 1 cm of continuous staining of the long axis and full circumference of the nerve [22]. After injection and dissection of one side, the cadaver was turned over and placed in lateral recumbency and the procedures were repeated. Additionally, the number of ribs, lumbar vertebrae and the disposition of the abdominal wall muscle layers were determined in each specimen. Both the clinician (IC) and anatomist (SK) were involved in counting positively stained nerve segments in each hemiabdomen.

### Statistical analysis

Staining of investigated segmental nerves was treated as a binary variable (positive/negative). Descriptive statistics were used to quantitatively describe and analyze the data. Results are presented as absolute numbers, median (range) and proportions.

## Results

### Phase I: Anatomical assessment

The pig assigned to anatomical dissection exhibited 15 thoracic and six lumbar vertebrae (Fig 1). At the level of the lateral TAP injection the EOM, IOM and TAM were present (Figs 1 and 2B). At the level of the caudal retrocostal TAP injection, the CTM, EOM, TAM and delicate parts of the IOM aponeurosis, running between EOM and TAM, were identified. The belly of the IOM was absent (Figs 1 and 2D).

Both in the caudal retrocostal and lateral injection point, ventral rami of thoracolumbar nerves were found running within the TAP. The ventral rami of Th_12_ (fourth-last), Th_13_ (third-last), Th_14_ (second-last) and Th_15_ (last) were evaluated (Fig 1C and 1D). The last thoracic nerve was found running in the lateral region away from caudal border of the last rib, which is different compared to the rest of the thoracic nerves (Fig 1D). The detectable lumbar nerves running in the TAP in the region of the lateral injection were the L_1_ (iliohypogastric) and L_2_ (ilioinguinal) nerve (Fig 1D). The L_1_ emerged as a longer ventrally directed main branch and one to two short branches dorsally-penetrating and innervating the IOM (Fig 1D). Ventral rami of L_3-6_ nerves anatomically representing the genitofemoral, cutaneous lateralis femoris, femoral and obturator nerves, were found to run deep in the hypaxial muscles forming a distinct neural plexus.

### Phase II: TAP injections and dye spread evaluation

Eight hemiabdomens of four Landrace pig cadavers, weighting 35.6 kg ± 1.5 kg (mean ± SD; ranging from 33.8 kg to 38 kg) and 12 weeks of age at the time of euthanasia were successfully scanned, injected and dissected. In all hemiabdomens, two well-defined methylene blue-stained areas were observed, which corresponded to each TAP injection (i.e., the lateral and caudal retrocostal TAP injection points; Fig 3). All injections were performed at the first attempt and no additional test dye was needed. No signs of perforation of the parietal peritoneum or intra-abdominal distribution of dye were observed. However, traces of the dye were found in more superficial fascial planes because of the reflux of the dye through the needle penetration pathway.

The caudal retrocostal TAP injections yielded a positive staining of the last, second-last, third-last and fourth-last medial branches of ventral rami of thoracic nerves in of seven out of eight (87.5%), eight out of eight (100%), seven out of eight (87.5%) and three out of eight (37%) of the cases, respectively. The lateral TAP injection resulted in positive staining of L_1_, L_2_ and L_3_ in eight out of eight (100%), five out of eight (62.5%) and one out of two (50%) cases, respectively (Table 1).

**Table 1:** Total number and proportions of positively (Y) or negatively (N) stained branches of thoracic and lumbar nerves after performing bilateral ultrasound-guided caudal retrocostal (blue) and lateral (red) transversus abdominis plane injections in four pig cadavers.

Pig No.	Hemi-abdo-men	Thoracic verte-brae No.	Lumbar verte-brae No.	Thoracic nerves				Lumbar nerves			No. stained:	No. stained:
				4^th^ last	3^rd^ last	2^nd^ last	last	L_1_	L_2_	L_3_	4^th^ last- L_2_ (max 6)	4^th^ last- L_2/3_ (max 6–7)
**1**	L	17	6	N	N	Y	Y	Y	Y	-	4	4/6
**1**	R			N	Y	Y	Y	Y	Y	-	5	5/6
**2**	L	16	6	N	Y	Y	N	Y	N	-	3	3/6
**2**	R			Y	Y	Y	Y	Y	N	-	5	5/6
**3**	L	15	6	Y	Y	Y	Y	Y	Y	-	6	6/6
**3**	R			N	Y	Y	Y	Y	N	-	4	4/6
**4**	L	16	7	N	Y	Y	Y	Y	Y	N	5	5/7
**4**	R			Y	Y	Y	Y	Y	Y	Y	6	7/7
**Positively stained (>1 cm)**				**3/8**	**7/8**	**8/8**	**7/8**	**8/8**	**5/8**	**1/2**		
**Median (range)**											**5 (3–6)**	**5 (3–6 (7))**

L, left; N, no; No., number; R, right; Y, yes.

The color grey indicates that the nerve was not identified in that specimen.

The approach combining lateral and caudal retrocostal injections with 0.3 mL/kg/injection site yielded a median of 5 (3–6) stained nerves from the fourth-last thoracic to the L_2_ nerve (a maximum of six nerves).

The thoracolumbar anatomy varied among individual animals from the phase II that showed 15 to 17 thoracic and 6 or 7 lumbar vertebrae, respectively (Table 1). Depending on the number of thoracic vertebrae in each animal thoracic nerves Th_12-14_ (fourth-last), Th_13-15_ (third-last), Th_14-16_ (second-last) and Th_15-17_ (last) were evaluated (Table 1). In the cadavers of phase II, the last thoracic nerve was found in the lateral region away from caudal border of the last rib. The detectable lumbar nerves running in the TAP in the region of the lateral injection were L_1-2_ (iliohypogastric and ilioinguinal nerve) or L_1-3_ (cranial-, caudal iliohypogastric and ilioinguinal nerve) in pigs with six or seven lumbar vertebrae, respectively (Fig 1D). Ventral rami of L_3-6_ in pigs with six and L_4-7_ in pigs with seven lumbar vertebrae, anatomically representing the genitofemoral, cutaneous lateralis femoris, femoral and obturator nerves, were found to run deep in the hypaxial muscles. In the pigs dissected in more detail to trace the origin of thoracolumbar nerves, a plexus formation could not be observed in neither the iliohypogastric nor the ilioinguinal nerves.

## Discussion

The injection of 0.3 mL/kg of dye through an US-guided caudal retrocostal and lateral approach for a TAP injection resulted in positive staining of a median of 5 (3–6) nerves responsible for the sensory innervation of the periumbilical and caudal abdominal wall in pig cadavers. Additionally, this study described the relevant anatomy of the abdominal wall of pigs. The TAP was found between different muscle layers in the described anatomical regions. In the caudal retrocostal approach and the lateral approach the TAP was found between the EOM and TAM, and between the IOM and TAM, respectively.

Ventral branches of the caudal thoracic and lumbar spinal nerves, which run within the TAP, provide somatosensory innervation to the lateral, ventro-lateral and ventral aspects of the caudal thoracic, cranial, umbilical and caudal abdominal regions in pigs [23–25]. A similar innervation pattern has been described in dogs [26]. Different from most other species is the highly variable number of thoracolumbar vertebrae in pigs. For instance, the thoracic vertebrae may vary from 13–18 [24, 25, 27–29] while the number of lumbar vertebrae range from 5–7 [25, 27, 29]. Although the pig cadavers included in this study belong to the same breed, the number of thoracic and lumbar vertebrae and subsequently, the number of ventral branches of the respective spinal nerves, differed among individuals (n = 15–17 and 6–7, respectively; Table 1). The varying number of vertebrae also affected the abdominal somatosensory innervation and is subject to genetic variation. It has been reported that the iliohypogastric nerve is absent in pigs with five lumbar vertebrae and its receptive field is presumably taken over by the costoabdominal (last thoracic) nerve [25]. In this case, the first lumbar segment provides a major part of its nerve fibers to the ilioinguinal nerve [25]. In pigs with six lumbar vertebrae the iliohypogastric nerve (L_1_) was present. In animals with seven lumbar vertebrae, the first two lumbar nerves represent the cranial (L_1_) and caudal (L_2_) iliohypogastric nerves, respectively [23, 25]. This may depend on the breed and individual differences within a breed, as documented here.

All the lumbar nerves, with or without intersegmental fiber exchange, can be involved in the formation of the lumbar plexus and thus in sensitization of the hind limb [24]. Interestingly, in the pigs dissected in more detail, neither L_1_, L_2_ nor L_3_ (ilioinguinal nerve in pig with 7 lumbar vertebrae) had involvement in the lumbar plexus.

Additionally, the anatomy of the abdominal wall in pigs is unique. In the paralumbar area the abdominal wall consists of EOM, IOM and TAM and sonographic references of the TAP in pigs are similar to humans, dogs and ponies [10, 13, 22]. However, in the caudal retrocostal area in pigs, the abdominal wall consists of a prominent CTM, EOM and TAM, as described in the present study. The belly of the IOM in the pig is very short and it ends on the last rib and does not reach the ventral midline and cranial abdomen (Fig 1C). Nevertheless, due to a prominent CTM, three muscle layers can be sono-anatomically recognized in the retrocostal area (Fig 2D). Furthermore, similar to humans, in swine species the IOM aponeurosis is assumed to split into two lamellae both involved in rectus sheath formation [30]. The dissection of the delicate IOM fascia in the caudal retrocostal area was not performed in this study. However, it is expected that in the retrocostal area delicate parts of the IOM aponeurosis, not ultrasonographically visible, lie in between EOM and TAM bellies forming the fascial TAP.

Both the lateral and caudal retrocostal TAP injection approaches in this study were conducted appropriately and without difficulties. Modifications of TAP block in order to desensitize the abdomen in various animals with their unique anatomical features have been suggested [14, 17–19, 21]. In this context, a two-point TAP block provides a wider coverage to reach nerves responsible for both the cranial and caudal abdominal wall. As an example, Portela et al. [14] and Teixeira et al. [17] used two injection points within the TAP plane in dogs aiming to increase the spread of LA and to provide better analgesia for the long abdominal incisions. Recently, Mirra et al. [21] also described a two-injection point TAP in calf cadavers. Due to the very long abdomen in pigs, we hypothesized that two injection points would be required to reach all the relevant nerves that provide innervation to the periumbilical and caudal abdominal wall. From the fourth-last thoracic nerve to the L_2_ (six nerves) median of 5 (3–6) nerves were stained per hemiabdomen. Altogether, our results provide evidence that two-injection point TAP block in pigs at the studied volume is needed in order to involve periumbilical and caudal abdominal wall. Nonetheless, the spread of dye obtained after the lateral TAP injection with given volume appeared sufficient to involve the nerves supplying caudal aspects of the abdominal wall.

The nerves stained with the caudal retrocostal TAP approach were the second-, third- and fourth-last thoracic nerves. These provide somatosensory supply to the abdominal wall around the umbilicus and cranial to it, and were positively stained in 8/8 (100%), 7/8 (87.5%) and 3/8 (37.5%) injections, respectively. Nerves cranial to the fourth-last thoracic spinal nerve were not stained. Surgical interventions involving cranial thoracic areas may require an additional cranial injection at the middle of the retrocostal area, close to the sternum, as it was described in Shetland ponies [18].

The nerves stained with the lateral approach involved in periumbilical, caudal and paralumbar abdominal wall innervation were the last thoracic nerve, and the first, second and third lumbar nerves. The last thoracic nerve (i.e. costoabdominal nerve), which was stained in 7/8 (87.5%) cases with the lateral approach, is the major nerve responsible for sensitization of periumbilical area. In hindsight, it might be advisable to inject LA into more cranial aspects of the paralumbar fossa when performing the lateral injection in order to involve the costoabdominal nerve, especially in pigs with five lumbar vertebrae as the last thoracic (costoabdominal nerve) becomes a major nerve for innervation of the caudal abdominal wall [23]. This could have been accomplished with different positioning of the ultrasound probe perpendicular to axis of the body close to the last rib and the in-plane technique. However, further studies would be needed to evaluate the proposed technique. The L_1_ (iliohypogastric nerve), which is a major nerve for caudal abdominal wall innervation [23–25] was positively stained in all 8/8 (100%) lateral injections. The L_2_, also crucial for caudal abdominal wall innervation in pigs was positively stained in 5/8 (62.5%) lateral injections. The high number of stained L_1_ and L_2_ nerves confirms the appropriate positioning of the US-probe and the volume used as appropriate. The short IOM which covers last thoracic, L_1_ and L_2_ nerves limits the cranial spread of the dye. This suggests, that using only lateral approach with the possibly higher volume of injectate might stain all the relevant nerves for desensitization of periumbilical and caudal abdomen. The L_3_ nerve was observed only in one pig with seven lumbar vertebra and was stained in one out of two hemiabdomens. Here, the L_3_ nerve corresponds to the ilioinguinal nerve, which is involved in the innervation of the caudal abdomen. We postulate that involvement of L_3_ in the TAP block is not necessary for the majority of procedures, except for the most caudal abdominal incisions. It could be possible that higher injection volumes are needed to involve L_3_ in the TAP block in animals with seven lumbar vertebrae. However, further studies are required to prove this assumption.

The possible paravertebral and epidural spread of dye after lateral injection was excluded by dissecting muscle layers towards the intervertebral foramina. No perforation of the parietal peritoneum or intra-abdominal distribution of dye was observed.

The TAP block relies on the large volume of injectate to cover several spinal nerves. The volume of the dye used (0.3 mL/kg/injection point) was based on previous similar studies in dogs [14, 17]. In general, distribution of LA in the TAP depends on the volume and the technique. The spread of tissue staining depends also on the properties of injected substance [31]. Moreover, the spread of LA in a clinical patient is expected to be similar though not the same as the distribution of the dye in cadavers due to breathing movements of the chest and abdominal wall, changes in the muscle tone, blood and lymph flow [11]. Additionally, potential toxicity and the effectiveness of different concentrations of LAs must be investigated before this technique is recommended for clinical use.

This pilot study has several limitations. First, the number of studied animals is low. Second, the scoring of the US-visualization of the target plane and of the technique performance was not conducted. Third, only one experienced anesthetist performed all the injections. Due to the cadaveric nature of the study, the potential efficacy of the injections could not be evaluated. Finally, due to the limited number of available animals, only one volume of the injectate was studied.

## Conclusion

The combined caudal retrocostal and lateral US-guided approach for a TAP injection in pig cadavers with 0.3 mL/kg/injection point of dye was feasible for an experienced operator. Consistent US visualization of the TAP was obtained. Medial branches of ventral rami of the spinal nerves from the fourth-last thoracic nerve to the L_2_ (n = 6) were stained with a median of 5 (3–6) nerves. Given that the degree of staining may be positively associated to nerve blockade, it is likely that the periumbilical and caudal abdominal wall would be desensitized in live animals. However, additional studies are required to assess the efficacy of this approach in pigs undergoing abdominal surgical procedures.
